# Special Issue “Novel Structural and Functional Material Properties Enabled by Nanocomposite Design” †

**DOI:** 10.3390/nano13030586

**Published:** 2023-02-01

**Authors:** Jürgen Eckert, Daniel Kiener

**Affiliations:** 1 Department of Materials Science, Montanuniversität Leoben, 8700 Leoben, Austria; 2 Erich Schmid Institute of Materials Science, Austrian Academy of Sciences, 8700 Leoben, Austria

Nanocomposites bear the potential to enable novel material properties that considerably exceed the capabilities of their individual constituent phases, thereby enabling the exploration of white areas on material property charts. In this inaugural Special Issue for the newly released subsection of Nanocomposites in the journal *Nanomaterials*, we aim to provide an overview of the current state of the art in enabling novel structural and functional material properties by presenting a better understanding and implementation of nanocomposite design. The covered properties of interest encompass the whole material usage span. This starts with structural modifications of nanocomposites by employing different synthesis routes, assesses their microstructure-dependent mechanical properties such as strength, ductility, and high-temperature stability. Furthermore, we address the functional characteristics of nanocomposites, such as soft magnetic properties or thermoelectricity, as well as the tailored property adjustment by design strategies (bioinspired design, chemical sensitivity, and bio sensing). Thus, the included contributions detail methods for the synthesis, characterization, modeling, and in-depth understanding of the mechanisms governing the outstanding properties of this fascinating material class.

In total, ten manuscripts including one review article, are published in this Special Issue, addressing the abovementioned characteristics and many more interesting aspects of nanocomposite materials. In the remainder, we present a brief overview of the some of the exciting insights of this Special Issue.

Petrinic et al. [1] synthesized citric-acid-covered superparamagnetic magnetite nanoparticles by co-precipitation for use as a draw solution agent on forward osmosis. The coated nanoparticles performed almost four-fold more strongly as an osmotic agent than the pure citric acid, making them a potential candidate for a broad range of concentration applications where current technologies are still limited.

In their study, Burtscher et al. [2] utilized severe plastic deformation to create a W–Cu nanocomposite. Through a combination of electron microscopy and elevated temperature nanoindentation experiments, they were able to assess the temperature-dependent elastic and plastic properties, including strain rate sensitivity and activation volumes, which are fundamental pre-requisites for future employment in extreme environments, such as fusion reactors.

Moon et al. [3] employed physical rather than chemical modifications of carbon-nanotube/polydimethylsiloxane composite electrode surfaces to enhance the detection of biomolecules such as DNA. By dip-coating the electrodes with functionalized multi-wall carbon nanotubes, the detection limit could be increased by a factor of more than 1000 compared with the original composite, paving the way towards lower detection limits of biosensor systems.

The report by Gao et al. [4] addresses the sintering bonding of SiC-particle-reinforced Al matrix composites using Cu nanoparticles and liquid Ga as self-fluxing fillers. Based on microstructural and mechanical analysis, they report that the metal matrix composites can be tuned with respect to joint strength and gas tightness by adjusting the Cu nanoparticles and the sintering temperature.

Gunti et al. [5] assessed the effect of different rolling conditions on the hardness and shear band revolution of the Virtreloy 1 bulk metallic glass using nanoindentation to different maximum load levels. The increase in pop-in events during nanoindentation decreased with the amount of cold rolling, indicating a more homogenous deformation of the metallic glass during rolling, which will benefit future forming operations of such materials.

In their study, Spinelli et al. [6] targeted the enhanced thermal conductivity of polylactic acid. They used graphene nanoplatelets as filler material in the polymer. By adding 9 wt% and ensuring favorable alignment of the filler platelets, the thermal conductivity increased by more than 250%, opening novel views towards heat transfer applications.

Wilmers et al. [7] studied the hierarchical morphology of the enameloid of two different shark teeth: biological hard and tough nanocomposite consisting almost entirely out of brittle phases. Analyzing the structural patterns in comparison to amniote enamel enabled the identification of microstructural design principles for ensuring certain biomechanical functions, thereby deriving strategies for the design of bioinspired composite materials with superior properties.

In the study by Wu et al. [8], different amounts of urushiol, extracted from raw lacquer and known for its acid-resisting properties, were added to a fixed amount of polyacrylonitrile, subsequently electrospun into nanoscale fibers, and deposited as thin films. The addition of urushiol improved the mechanical strength and chemical stability, rendering the fibers unique materials with strong acid resistance and weak acid dissolution properties for future use.

Shuleiko et al. [9] used femtosecond laser machining to introduce nanoscale one-dimensional laser-induced periodic surface structures on amorphous hydrogenated silicon. Furthermore, the laser treatment caused partial crystallization of the amorphous substrate into nanocrystalline Si. Taken together, this material surface modification strongly enables enhanced conductivity and introduces an orientation dependence.

The review by Kubenova et al. [10] focused on the thermoelectric properties of copper chalcogenides, with an emphasis on property modifications by substitution with sodium and lithium alkali metals. The cationic sub-lattice is responsible for the very low thermal conductivity, whereas nonstoichiometric defects provide high electronic conductivity; the combination of these aspects contributes to the high thermoelectric figure of merit, approaching a value of three.

## References

[1] Petrinic I., Stergar J., Bukšek H., Drofenik M., Gyergyek S., Hélix-Nielsen C., Ban I. (2021). Superparamagnetic Fe_3_O_4_@CA Nanoparticles and Their Potential as Draw Solution Agents in Forward Osmosis. Nanomaterials.

[2] Burtscher M., Zhao M., Kappacher J., Leitner A., Wurmshuber M., Pfeifenberger M., Maier-Kiener V., Kiener D. (2021). High-Temperature Nanoindentation of an Advanced Nano-Crystalline W/Cu Composite. Nanomaterials.

[3] Moon J., Jiang H., Lee E.-C. (2021). Physical Surface Modification of Carbon-Nanotube/Polydimethylsiloxane Composite Electrodes for High-Sensitivity DNA Detection. Nanomaterials.

[4] Gao Z., Yin C., Cheng D., Feng J., He P., Niu J., Brnic J. (2021). Sintering Bonding of SiC Particulate Reinforced Aluminum Metal Matrix Composites by Using Cu Nanoparticles and Liquid Ga in Air. Nanomaterials.

[5] Gunti A., Jana P., Lee M.-H., Das J. (2021). Effect of Cold Rolling on the Evolution of Shear Bands and Nanoindentation Hardness in Zr_41.2_Ti_13.8_Cu_12.5_Ni_10_Be_22.5_ Bulk Metallic Glass. Nanomaterials.

[6] Spinelli G., Guarini R., Kotsilkova R., Ivanov E., Romano V. (2021). Experimental, Theoretical and Simulation Studies on the Thermal Behavior of PLA-Based Nanocomposites Reinforced with Different Carbonaceous Fillers. Nanomaterials.

[7] Wilmers J., Waldron M., Bargmann S. (2021). Hierarchical Microstructure of Tooth Enameloid in Two Lamniform Shark Species, *Carcharias taurus* and *Isurus oxyrinchus*. Nanomaterials.

[8] Wu K., Shiu B.-C., Zhang D., Shen Z., Liu M., Lin Q. (2021). Preparation of Nanoscale Urushiol/PAN Films to Evaluate Their Acid Resistance and Protection of Functional PVP Films. Nanomaterials.

[9] Shuleiko D., Martyshov M., Amasev D., Presnov D., Zabotnov S., Golovan L., Kazanskii A., Kashkarov P. (2021). Fabricating Femtosecond Laser-Induced Periodic Surface Structures with Electrophysical Anisotropy on Amorphous Silicon. Nanomaterials.

[10] Kubenova M.M., Kuterbekov K.A., Balapanov M.K., Ishembetov R.K., Kabyshev A.M., Bekmyrza K.Z. (2021). Some Thermoelectric Phenomena in Copper Chalcogenides Replaced by Lithium and Sodium Alkaline Metals. Nanomaterials.

